# Two years of COVID-19 pandemic in Jordan: A focus on epidemiology and vaccination

**DOI:** 10.7189/jogh.12.03063

**Published:** 2022-10-01

**Authors:** Arwa Qaqish, Mariam Al-Omari, Manal M Abbas, Mahmoud Ghazo

**Affiliations:** 1 Department of Biology and Biotechnology, Faculty of Science, The Hashemite University, Zarqa, Jordan; 2 Department of Basic Medical Sciences, Faculty of Medicine, Yarmouk University, Irbid, Jordan; 3 Department of Medical Laboratory Sciences, Faculty of Allied Medical Sciences, Al-Ahliyya Amman University, Amman, Jordan; 4 Pharmacological and Diagnostic Research Lab, Al-Ahliyya Amman University, Amman, Jordan; 5 Jordan Ministry of Health, Laboratory Directorate, Amman, Jordan

Jordan’s experience with COVID-19 has gone through several phases varying from limited transmission to a cluster of cases to widespread dissemination starting from March 2020 until now [1,2]. Here, we aimed at narrating the story of COVID-19 spread in Jordan from the beginning. We gathered data from published articles, Ministry of Health (MOH) and WHO websites, data tracker and Jordanian news websites, to be all put together into a single article that would document major events of COVID-19 spread and vaccination in the country.

## MARCH TO AUGUST 2020: THE BEGINNING OF COVID-19 SPREAD IN JORDAN, A MODEL OF SUCCESSFUL CONTROL

A case coming from Italy registered the first record of COVID-19 in the country on March 2, 2020 [3]. Afterwards, cases started to increase in a relatively slow rate compared to most countries. By the end of August, Jordan had less than 2000 cases and 25 deaths. Afterwards, number of confirmed cases increased dramatically.

The early response to COVID-19 epidemic in Jordan was viewed as a promising approach [3]. The low number of incidences from March to the end of August 2020 was mainly attributed to the preventative measures enforced by the Jordanian government to help contain viral spread. Limitation of movement, strict curfew, reduced working hours, working from home when possible, distant learning, prohibition of gatherings of more than 20 people, suspension of international flights and a total lockdown that started in March 14 and continued for several weeks; all made Jordan a role model in fighting the deadly pandemic. However, these measures deeply impacted the kingdom’s fragile economy and caused human suffering.

### September 2020 to January 2021: first wave of viral spread

The unavoidable spread of the virus at the community level started in or before September and peaked in November 2020 as the first wave of COVID-19 in the country, resulting in an accumulative of 170 000 cases and 2000 deaths. Early in September, land boundaries were opened for trucks importing goods from neighbouring countries. Truck drivers could mingle with the population without taking quarantine measures seriously. This seemed to correlate strongly with the drastic change in the number of confirmed cases and deaths. Concurrently, the “in class learning” school year started, and universities opened doors for registration. People started to get less serious about social distancing and face covering and, some myths about COVID-19 to be nonexistent disseminated among the population [4].

This first wave was dominated by two Jordanian lineages, namely (B.1.1.312 and B.1.36.10) [5]. Molecular analyses also revealed that a UK variant of concern was introduced into the country from November 2020 and became the dominant lineage from the new year 2021 onwards [5].

### January to May 2021: second wave of viral spread

**Figure 1:**
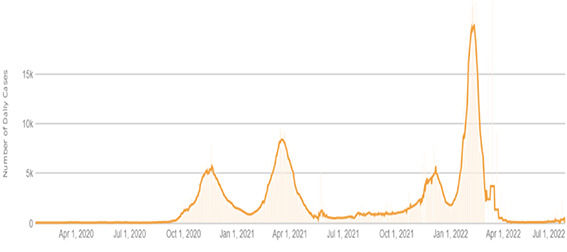
Overview of COVID-19 in Jordan by date. The figure was adapted from COVID-19 Dashboard by the Center for Systems Science and Engineering (CSSE) at Johns Hopkins University [6].

The fast-spreading UK variant probably resulted in the more aggressive second wave of COVID-19 transmission that started late January and peaked in mid-March 2021 [1,5]. This wave started to decline progressively with the end of March. By the end of June 2021, daily case records reached an approximate of 500 compared to >9500 cases during peak days (**Figure 1**).

The first 3 cases of infection with the Indian delta strain were reported on May 1, 2021 [7]. These were Jordanian residents who did not travel. By June 23, 2021, 211 people recorded infections with mutated variants of coronavirus, 170 of whom were diagnosed with a new COVID-19 strain called “Delta Plus” [8]. By July 6, 2021, 245 infections with the super-contagious Delta Plus were reported; 222 in the capital Amman and the rest in Mafraq, Zarqa, and Karak [9].

Despite of the introduction of the fast-spreading delta strain, the epidemiologic curve remained somehow stable [1,2]. This flattening of the epidemiologic curve might have resulted either from naturally acquired or vaccine induced immunity against the virus.

Due to the economic stress imposed on the nation, restrictions on all sectors and curfews were removed with supervision of mask covering, social distancing and proof of vaccination [10]. On September 1st, the school year started for total in-class education. It was mandatory for all teachers and staff to have received 2 shots of vaccine. Seating arrangements ensured distancing between students, and facial masks were obligatory for all. Simultaneously, the Ministry of Culture and Tourism held several festivals and singing concerts. In the mid of October, universities opened doors under the obligation of proof of full vaccination for staff and students, or a negative PCR testing report every 3 days [11].

Positive cases were reported among school students. Asymptomatic infection is very likely to occur within children and young teenagers. These cases might have been a major cause for the emergence of the third wave that hit the country starting mid-October 2021.

### October 2021 to January 2022: third wave of viral spread

A third wave of viral spread blasted off in mid-October 2021. The number of daily reported cases peaked on December 8 to reach >6000 cases, and started to decline progressively afterwards [1,2]. Noticeably, the third wave is considerably less aggressive compared to the second wave. This could have been a result of the continuous efforts of active COVID-19 vaccination driven by the MOH.

On December 9, 2021, Jordan announced the detection of two cases infected with the Omicron variant. The first is a Jordanian national who recently returned from South Africa and the second is a Jordanian national who has not left the country. This raised fears that Omicron may be spreading within the population [12]. One week later, five cases harbouring the variant, all from abroad, were reported in the Kingdom, all were put under institutional quarantine [13]. By the end of the year 2021, the number of Omicron cases reached >300 [14].

**Figure:**
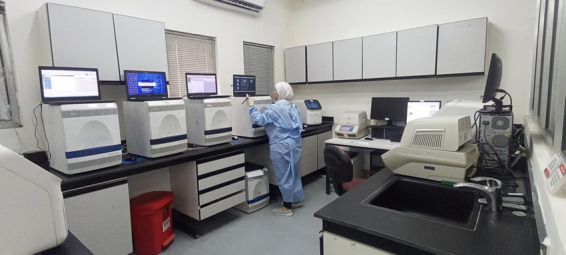
Photo: Expanded capacity of PCR testing of COVID-19 at the Central Public Health Laboratory (CPHL) in the capital Amman as a governmental response to manage the pandemic. Source: From the authors’ own collection. Used with permission.

### January to March 2022: fourth wave of viral spread

Early January 2022 witnessed the launching of the fourth wave of COVID-19 spread. This wave was described to be embedded within the previous wave. On January 20, the omicron mutant accounted for 55% of new infections [15]. By mid-February, infections escalated vigorously to reach a peak of ~ 30 000 per day, an approximate 30% of tested samples. The caseload started a downward trajectory afterwards [1,2]. Despite its fast spread, Omicron proved to cause minimal symptoms associated with the upper respiratory tract, limiting the need for hospital admissions [15].

After mid-February 2020, in hopes of reviving the economy, the government made several announcements to gradually lift strict regulations related to COVID-19 management. The government announced the reduction of the isolation period for infected people to five days starting from the date of + ve PCR testing, without the need for another test after the isolation period. The government also decided to cancel the isolation period for contacts of infected individuals. Additionally, those coming to Jordan no longer needed to conduct a PCR test upon arrival. PCR testing will not be required for attending events, given proof of full vaccination. Schools and classrooms will no longer switch to online learning in case of a 10% infection rate. On March 1, 2022, MOH ceased to give daily reports on the number of COVID-19 infections and deaths. Instead, a weekly Sunday Report is announced in the news [16].

By the time of writing this viewpoint, July 12, 2022, the number of cases reached a total of confirmed cases 1 702 661 and 14 069 deaths (**Table 1**, **Figure 1**) [1,2].

**Table 1:** The accumulative number of confirmed cases infected with COVID-19 in Jordan appearing by end of each wave and the accumulative number of fully vaccinated individuals

Wave number	Number of confirmed cases	Number of confirmed deaths	Number of fully vaccinated personnel
First wave November 19, 2020	169 395	2053	
Second wave March 15, 2021	486 470	5428	48 379
Third wave January 16, 2022	1 100 967	12 986	4 109 437
Fourth wave March 18, 2022	1 689 314	14 003	4 425 683
Total number July 12, 2022	**1 702 661**	**14 069**	**4 544 593**

### COVID-19 vaccination in Jordan

Jordan launched its COVID-19 vaccination program in January 2021. MOH invited all residents of Jordan, including refugees, to register for free vaccination [17].

Vaccination in Jordan faced hard challenges related to the acceptance of the population to receive vaccines of any type. Rumours around deleterious side effects such as infertility, belief in conspiracy theories and generalized anti-vaccine trends all resulted in a state of vaccine hesitancy [18]. Among university students, considered a knowledgeable group, the intention to get vaccinated was low (34.9%) [18]. Similarly, considering the general Jordanian community, the acceptance rates for COVID-19 vaccines was 29.4%. Vaccine hesitancy was found to be mainly associated with dependence on social media platforms as source of information instead of medical doctors and scientists [19].

Due to the global urgency of the pandemic, many COVID-19 vaccines were developed and authorized for use in a relatively short time. This created many doubts around the efficacy and safety of these vaccines. A study performed by a team in the Hashemite University of Jordan aimed to assess the side effects and perceptions following COVID-19 vaccination in the country [17]. This study revealed that most vaccine side effects were mild, common, and non-life-threatening. Interestingly, more than half of the of participants were hesitant and anxious before vaccination; but around 95% of them have advised others to get vaccinated afterwards, confirming that getting vaccinated makes people more reassured [17].

Four vaccines have been approved and used so far in Jordan: Pfizer-BioNTech, Oxford-AstraZeneca, Sinopharm (Beijing) and Sputnik V (Gamaleya). Typically, the vaccine is given in two doses, separated by a window of at least 3 weeks depending on vaccine type. November 13, 2021, MOH announced the availability of a third vaccine dose to those aged 18 and above after at least six months of receiving the second dose. The ministry stated that it is preferred that the third dose be of the same type as the first and second doses unless the type of vaccine taken affected the person with adverse effects [20].

With the return of school children to in-class learning for the second semester, The Ministry of Education, resumed a campaign that started on December 2021-January 2022 for the vaccination of students from 7th to 12th grade at public schools across the Kingdom, given parents’ written approvals [21].

Up to the time of writing this article, the number of fully vaccinated people in the country reached 4 544 593, which forms around 50% of the total population (**Table 1**) [2].

It is not known yet whether the percent of immune protected individuals, by natural infection and vaccination, is reaching the near 60 to 70% protection rate typical of herd immunity in a population. Still, the continuous emergence of new variants of the virus is a concern that threatens immune protection vaccination strategies.

### Experiences that Jordanians acquired from the pandemic

We hope that many lessons have been learned from these 2 years of pandemic experience. The Jordanians lived an era of infectious disease spread. They learned to acquire necessary information from trusted sources such as the MOH, not put confidence in the social media and hopefully started to doubt the conspiracy theory. They learned how to protect themselves and loved ones from infection by following preventative measures imposed by the government. Jordanians also experienced vaccination regimens and hopefully the vaccine hesitancy barrier was broken, at least partially. Finally, in terms of education, for the first time in Jordan, online platforms were created, giving a chance for potential online education in the future as applied in many developed parts of the world.
